# Preventive Dentistry

**Published:** 1895-05

**Authors:** I. P. Wilson

**Affiliations:** Burlington, Iowa.


ARTICLE IV.

### PREVENTIVE DENTISTRY.

BY I. P. WILSON, D. D. S., BURLINGTON, IOWA.

That very many of the operations performed in a dental practice could have been avoided, had the teeth been properly cared for, there can be no doubt.

The masses of people do not understand the importance of hygienic dentistry. Many, who observe the most scrupulous care as to personal cleanliness, and are even fastidious as to what they will eat and wear, and religiously guard every avenue of approach for disease that surrounds their homes or their places of business, yet they tolerate offensive roots or stumps of teeth in their gums, with tartar and decomposed food upon their teeth. These impurities vitiate the air that they breathe and poison the food that they eat in the first process of digestion, and thereby render impure the blood which must supply the continued demand made upon it for reparative material.

The masses of people need to be educated regarding this all important matter. Who shall be the teachers? Were it not that parents, as a rule, are ignorant of the consequences of the neglect referred to above, we might excuse ourselves, and leave the rising generation in the hands of fathers and mothers. But we cannot, as a profession, afford to do this.

In the first place a dentist should set an example of cleanliness. His teeth should be scrupulously clean, and his breath pure. He should not hesitate to speak frankly and freely to his patients regarding a personal matter that has so much to do with health, not only of the dental organs, but of the entire organism. Sensible people will appreciate such advice and instructions, while others who are more thoughtless, I had better said more senseless, will perhaps manifest indifference, and they may give you to understand that they do not appreciate any gratuitous advice, as they may deem it, pertaining to their personal habits.

The physician or the dentist who will stand by and see his patient's health, if not his life threatened with danger, and not fearlessly sound the alarm, is not worthy of his high calling.

Not only do the interests of our patients demand instruction at our hands, but our personal interests demand it.

It is the privilege of every dental practitioner to build up a clean, desirable practice that will not give him discomfort, nor endanger his health. Let me emphasize more fully what I would say with an illustration:

Mrs. A. requests you to kill the nerve, treat and fill an aching tooth. On examination you find her teeth coated with tartar and decomposed food. You find some dead useless roots or stumps of teeth in her mouth, you also find that a number of her teeth are decayed and need filling, and, as a matter of course, you find Mrs. A. with an exceedingly unpleasant breath, and by no means a desirable patient upon whom to operate. You very properly suggest that a general renovation is necessary; that after an application has been made to devitalize the pulp, and relieve her of pain, that her teeth must next be cleansed, and then *kept* clean. The necrosed roots must be extracted, and the decayed teeth filled.

If this course of treatment is objected to by the patient, freedom from pain being the only object sought, I should promptly decline treating and filling said tooth, having too much respect for my olfactory nerves, as well as my precious health to voluntarily place myself under such contaminating influences.

It seems to me that in such cases our duty is plain. We cannot afford to jeopardize our own health, nor is it right that we should encourage human beings in totally disregarding hygienic laws. This may seem like heroic treatment, but nothing else will sufficiently arouse some people, and awaken their self-respect and pride, so as to stimulate them to action.

But I wish now to call attention to the thought that was first in my mind when I sat down to write this short paper.

It is a well known fact that, as a rule, the teeth of boys and girls decay more readily during their teens than in subsequent years. The reason is obvious, and need not be dwelt upon here. The preventive treatment I have to recommend is simple and effective, especially in the bicuspid and molar teeth. When these teeth first emerge from the gums, the enamel fissures are open, and a fine pointed excavator can readily be forced down into the immature dentine.

The imperfectly developed enamel in these fissures is sometimes mistaken for caries, and is treated accordingly.

Instead of attempting excavation in such cases, let the fissures be thoroughly syringed out with warm water, then dehydrate with alcohol, dry thoroughly, and fill with cement. Then place a little of the dry powder over the filling and press it firmly with the finger, forcing the cement into the remotest recesses of the fissures, and excluding the moisture, if necessary, with the finger still upon the filling, and holding it there until crystallization has taken place. Then dress off the surplus, and in nine cases out of ten decay has been prevented, and without causing any pain from excavation.

But if decay has been prevented for only a few months or years, and the same treatment is then repeated, and the tooth is preserved through those early years of immaturity, and is afterward filled with gold, more of the tooth structure has been preserved, its natural color retained, and much less pain has been inflicted.

I need not enumerate the different conditions of the teeth, particularly in youth, and in pregnant and nursing women where cement may be used to great advantage in preventing decay.

Preventive dentistry should be practiced more by the profession than it is, and our patients will not be slow to appreciate our efforts, and remunerate us more willingly for preventive treatment, than for more taxing services.—*Dental Digest.*

---

ARTICLE V.

